# Clinical efficacy analysis of anterior superior iliac spine 3D-printed guide plate-assisted sacroiliac screw placement for the treatment of pelvic fractures

**DOI:** 10.3389/fsurg.2025.1644194

**Published:** 2025-11-05

**Authors:** Lin Chen, Lan Luo, Xulin Zhang, Lei Li, Dong Liu

**Affiliations:** 1 Traumatic Orthopedics, Suining Central Hospital, Suining, Sichuan, China; 2 Gastroenterology Department, Suining Central Hospital, Suining, Sichuan, China; 3 Hand Micrographic Surgery, Suining Central Hospital, Suining, Sichuan, China

**Keywords:** pelvic fracture, sacroiliac screw, 3D-printed guide plate, fracture, 3D printed

## Abstract

**Objective:**

The aim of this study was to compare the clinical outcomes of percutaneous 3D-printed guides in the anterior superior iliac spine site with traditional fluoroscopy-assisted sacroiliac screw implantation for the treatment of pelvic fractures.

**Methods:**

In total, 40 patients with eligible pelvic fractures who were diagnosed and treated between December 2022 and May 2024 were enrolled and divided into two groups. The guide plate group used anterior superior iliac spine 3D-printed guide plate-assisted sacroiliac screws to fix the posterior pelvic ring fracture; the fluoroscopy group used fluoroscopic freehand placement of sacroiliac screws to fix the posterior pelvic ring fracture. The operative time, number of fluoroscopies, amount of intraoperative bleeding, and length of incision for each screw implantation in the two groups were recorded. Fracture reduction was evaluated according to Matta’s criteria, screw position was assessed using modified Gras classification, and a statistical analysis was performed.

**Results:**

Among the 40 patients who were followed up for 6–12 months, 23 sacroiliac screws were implanted in 20 patients in the guide plate group and 25 sacroiliac screws were implanted in 20 patients in the fluoroscopy group. The operative times were 50–75 (60.00 ± 8.429) min and 50–90 (72.25 ± 10.939) min (*P* < 0.001) in the guide plate group and fluoroscopy group, respectively, while the number of fluoroscopies was 7–18 (11.65 ± 3.117) and 38–62 (48.05 ± 7.258), respectively (*P* < 0.001), and the amount of intraoperative bleeding was 10–30 (16.60 ± 5.642) mL and 5–20 (10.3 ± 4.354) mL, respectively (*P* < 0.001). Matta’s criteria scores for fracture repositioning in the two groups were excellent and good, respectively (*P* = 0.429). Modified Gras classification was used to assess the screw positions, with 19 in class I, 4 in class II, and 0 in class III in the guide plate group and 20 in class I, 4 in class II, and 1 in class III in the fluoroscopy group. The difference between the two groups was not statistically significant (*P* = 0.624).

**Conclusion:**

Anterior superior iliac spine 3D-printed guide plate-assisted sacroiliac screw fixation of pelvic fracture, when compared with traditional fluoroscopy-assisted sacroiliac screw fixation of pelvic fracture, has a shorter operation time, reduces the number of fluoroscopies, and improves the accuracy of implantation.

## 1 Introduction

In recent years, with the improvement of social and economic levels, more patients with pelvic fractures are encountered in clinical diagnosis and treatment, accounting for approximately 3% of systemic fractures (1). Pelvic fractures are often caused by high fall injuries, traffic accidents, and other major traumas, and therefore are usually combined with other injuries, with high complication and mortality rates (2, 3). The posterior pelvic ring accounts for 60% of the overall stability of the pelvis, and therefore, fracture of the posterior ring is the main factor leading to instability of the pelvic ring (4, 5), and the incidence of unstable fractures is 17%–30% (6). Reduction and fixation of the anterior and posterior pelvic rings is currently the gold standard for pelvic fracture treatment (7). The common surgical methods for posterior pelvic ring fractures are internal fixation with self-cutting plate screws and internal fixation with cannulated screws, both of which can achieve satisfactory reduction and functional activity requirements and have the same clinical results. Due to the special anatomical position of the pelvis and the concentration of blood vessels and nerves around the pelvis, the surgical incision and reduction process requires extensive stripping and can lead to intraoperative bleeding and relatively more postoperative complications. Moreover, due to individual differences in the anatomy of the pelvis, there is no uniform shaping plate, and during the surgical process, it is necessary to shape the plate and perform other operations, which increases the surgical time and the number of fluoroscopies. Minimizing the difficulty of the surgery and reducing the occurrence of medical complications while performing the reduction and fixation of the fracture is currently a research focus (1).

The sacroiliac screw is suitable for percutaneous implantation and has good biomechanical properties. In addition, in percutaneous fixation, it has the advantages of requiring a shorter operation time and leading to less bleeding and less soft tissue damage (8, 9). However, it has high technical requirements. The incidence of inaccurate sacroiliac screw position under conventional x-ray fluoroscopy during freehand percutaneous placement of sacroiliac screws is 2%–15%, and the incidence of asymptomatic lumbosacral nerve and iliac vessel injury is 0.5%–7% (10). Thus, the main reason for the difficulty of sacroiliac screw placement is the complexity of the adjacent soft tissue structures. In this regard, how to accurately insert sacroiliac screws into the sacrum and ensure their effective stabilization is an urgent problem to be solved and optimized (11).

Some scholars have proposed the use of computer navigation systems to assist the placement of sacroiliac screws, which could greatly improve the safety and accuracy of the operation, and truly realize the ideal of minimally invasive surgery, reflecting precision medical treatment. However, at present, the cost of computer navigation-assisted surgery is high, which makes it difficult for general hospitals to afford, and patients also find it difficult to bear the expensive medical costs, which are not conducive to the popularization of this technology (12). With the widespread use of 3D printing technology, 3D-printed guides are increasingly being used to assist in the minimally invasive placement of sacroiliac screws, with less intraoperative bleeding, accurate nail placement, fewer postoperative complications, and precise efficacy. Most hospitals are able to conduct 3D printing work, and the price is moderate, which hospitals and patients can accept, leading to clinical popularization.

Posterior ring fractures alone are rare in pelvic fractures, and posterior ring fractures are usually combined with anterior ring fractures. Therefore, for most of the pelvic fractures, the anterior ring should be fixed at the same time as the posterior ring. In the past, 3D-printed pelvic guides were more commonly used as posterior iliac bone guides, which required stripping more soft tissues in the sacro-hip area in the prone position during surgery, and then changing to the supine position when fixing the anterior ring, which increased the surgical time and surgical difficulty to a certain extent. Based on this, our team designed a personalized anterior superior iliac spine 3D-printed guide plate to assist in sacroiliac screw placement, so that the anterior pelvic ring and posterior pelvic ring fixation can be performed simultaneously under supine conditions. In this study, the clinical efficacy of the new 3D-printed guide plate-assisted sacroiliac screw fixation technique was compared with the traditional placement of sacroiliac screws, and it will provide a reference for further clinical applications.

## 2 Data and methods

### 2.1 General data

The clinical data of patients hospitalized in our traumatology and orthopedics ward because of a pelvic fracture from December 2022 to May 2024 were retrospectively analyzed, and according to the inclusion and exclusion criteria, 40 patients were included in this study. According to the different treatment methods, the patients were divided into two groups: 20 cases in the experimental group who were fixed with the aid of a 3D-printed guide plate in the anterior superior iliac spine (Observation group) and 20 cases in the control group who were fixed without this guide using the C-arm fluoroscopy technique (Control group). The patients were followed up for more than 6 months after the operation, and one patient was lost. The comparison of the basic conditions of the patients is shown in Table 1, and the differences between the two groups were not statistically significant in terms of gender, age, BMI, preoperative waiting time, side of the injury, fracture type (13), cause of injury, and the number of screws inserted (*P* > 0.05). The study protocol was approved by the ethics committee of the hospital (LLSLH20220091), and all the enrolled patients signed the relevant informed consent form.

**Table 1:** General characteristics of the participants.

Variable	Observation	Control	*χ*^2^/t	*P*
Male	7	8		
Female	13	12	0.107	0.744
Age	52.40 ± 12.684	56.45 ± 8.947	−1.167	0.251
BMI	22.54 ± 1.487	23.09 ± 2.278	−0.911	0.368
Waiting days before the operation	5.50 ± 1.960	5.80 ± 1.824	−0.501	0.619
Injury side: left Injury side: right	13	9		
	7	11	1.616	0.204
Type: Tile B	17	15		
Type: Tile C	3	5	0.625	0.429
Cause of injury: C	12	13		
Cause of injury: G	4	4		
Cause of injury: Z	4	3	0.183	0.913
Number of screws	23	25	0.944	0.624

Cause of injury: C, traffic accident injury; G, falling injury from a height; Z, heavy objects.

### 2.2 Inclusion and exclusion criteria

The inclusion criteria were as follows: (1) age 18–70 years old, (2) fresh unstable pelvic fracture, (3) can be fixed by closed reduction using sacroiliac screws, and (4) postoperative follow-up cycle of more than 6 months.

The exclusion criteria were as follows: (1) old pelvic fracture; (2) open pelvic fracture; (3) patients with nerve injury; (4) severe osteoporosis; (5) combined with severe heart, liver, and kidney function damage; severe coagulation dysfunction; malignant tumor; or obvious drug contraindications.

### 2.3 Surgical approach

#### 2.3.1 Preoperative preparation

After admission, according to the general condition of the patient, the relevant auxiliary examinations and tests were conducted, including pelvic CT + 3D reconstruction. Moreover, cardiac and electrical monitoring were conducted, with fluid replenishment, analgesic or anticoagulation medication, and femoral condylar traction administered if necessary. Finally, the surgical treatment was performed at an early stage after exclusion of contraindications to surgery.

#### 2.3.2 Anterior superior iliac spine 3D-printed guide plate preparation

**Figure 1:**
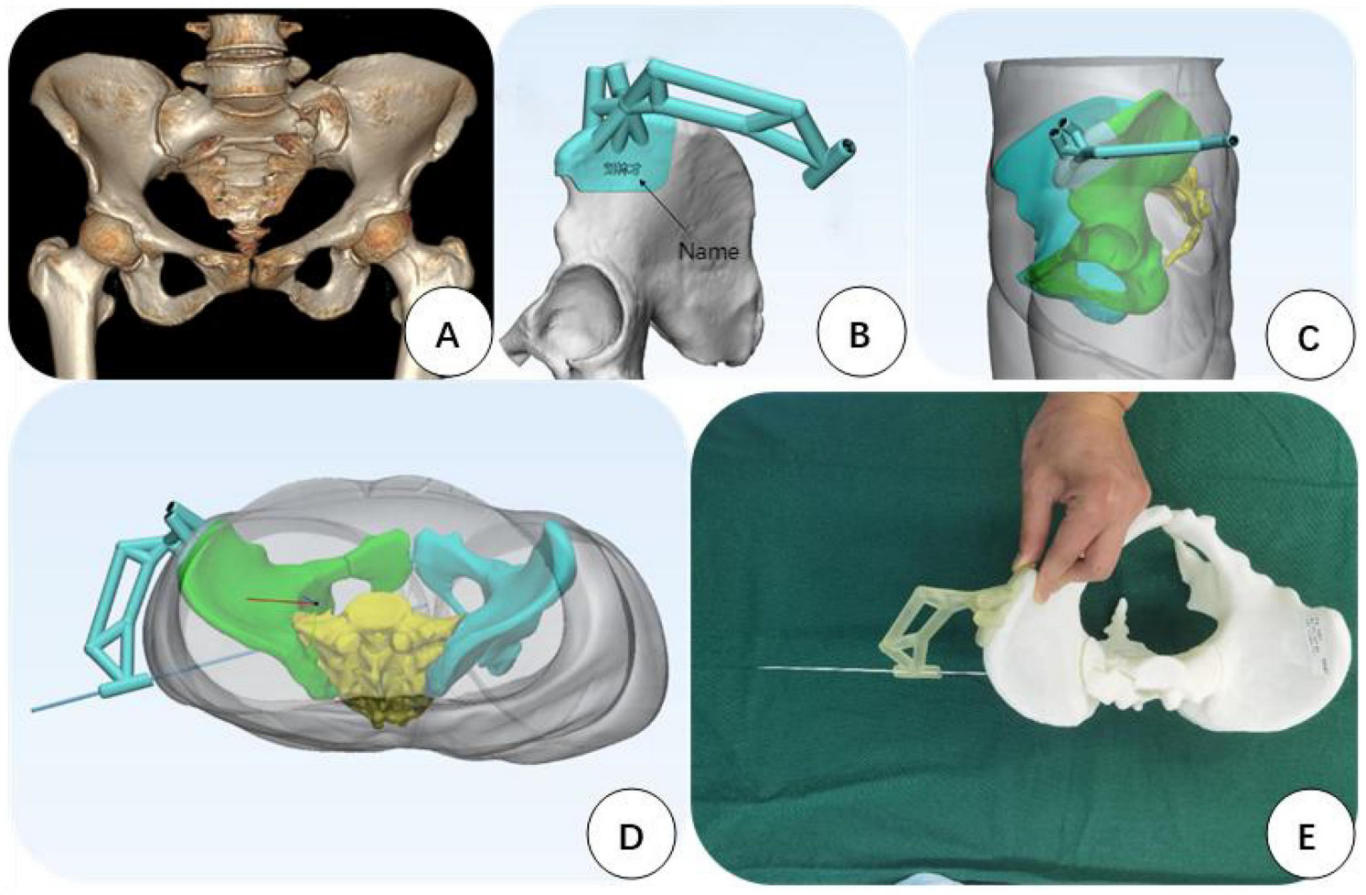
The design of the guide plate. **(A)** Preoperative CT reconstruction of the patient. **(B)** 3D-printed guide plate designed after importing the patient's CT data into the system. The base of the guide plate is located in the anterior superior iliac spine area and is adhered to the surface of the bone. **(C)** A connecting grasping device designed after reconstructing the patient's skin and soft tissues is located just on the skin surface. **(D)** Simulated insertion of the sacroiliac screws through the hollow guiding post, so that the screws are completely located in the bone. **(E)** Preoperative simulation of the guide wire process to check the safety of the guide plate.

The CT + 3D reconstruction data of the pelvis collected after the patient's admission were imported into Mimics 12.0 software to construct a 3D model of the pelvis. Utilizing the patient's shallow anterior superior iliac spine with specific anatomical landmarks and other characteristics, the base of the guide plate was made in the anterior superior iliac spine, with the top of the base close to the anterior superior iliac spine and shaped along the iliac spine to the posterior superior iliac spine and the outer side of the base close to the iliac outer plate. Thus, the guide plate was designed according to the actual size of the pelvis to make the base of the guide plate closely adhere to the surface of the bone. On the upper surface of the base, two 2.5 mm hollow channels were left in the iliac spine, so that two 2.5 mm Kirschner pins could pass through the channels and be used as the fixation pins for the guide plate. These fixation pins (Kirschner pins) serve the same role as the pins used in traditional external pelvic stents, which are external devices used in some surgical procedures to support or hold parts of the pelvis in place temporarily. The curved connecting device of the guide plate was designed using the patient's combined skin and soft tissue data and was designed to fit tightly on the skin surface. The steps to 3D printing the guide plate are as follows. First, virtualize a hollow channel with a diameter of 6.5 mm as a hollow screw channel on the pelvis model in the CT software, and adjust the direction of the channel in the inlet position, outlet position, and lateral position, so as to optimize the position of the screw channel. Then, extend the system outside the ilium bone to combine it with the connecting device, and make a cylindrical hollow guide post outside the skin. Then, import the patients’ data into the 3D printer (Xunshi Technology's UV-curable 3D Printer SprintRay Pro95), use photosensitive resin material (rb0808) to print the guide plate, and use the pelvic model to verify the correctness of the 3D-printed guide plate. Finally, use subcooling sterilization before the surgery to prepare the guide plate for use. The guide plate design ideas are shown in Figure 1.

#### 2.3.3 Surgical procedures

The Control group was routinely disinfected and required surgical draping of the lower abdomen and hip on the injured side for the C-arm fluoroscopy in the inlet position, outlet position, and lateral position to determine the 2.5 mm Kirschner's guide wire’s point of entry and the direction of the needle guide. The repeated 'intraoperative fluoroscopy determined when the Kirschner's guide wire reached the desired depth'. This was followed by drilling holes along the Kirschner's guide wire, 'into which the 6.5 mm cannulated screws were screwed. Kirschner's needle was then removed, and the wound was washed and sutured. Then, the Internal Fixation System (INFIX, which is a minimally invasive method used for treating unstable pelvic fractures) bracket was used to fix the anterior pelvic ring. An incision of approximately 2 cm was made approximately 2 cm below the bilateral anterior superior spine; the soft tissues were carefully dissected, the bone surface was exposed, and the iliac screw was inserted along the direction of the LC II (lateral compression fractures). The length of the transverse bar was pre-measured and shaped to fit the required dimensions, and then the transverse bar was inserted and fixed through the subcutaneous tunnel (14). Finally, the wounds were irrigated and sutured.

**Figure 2:**
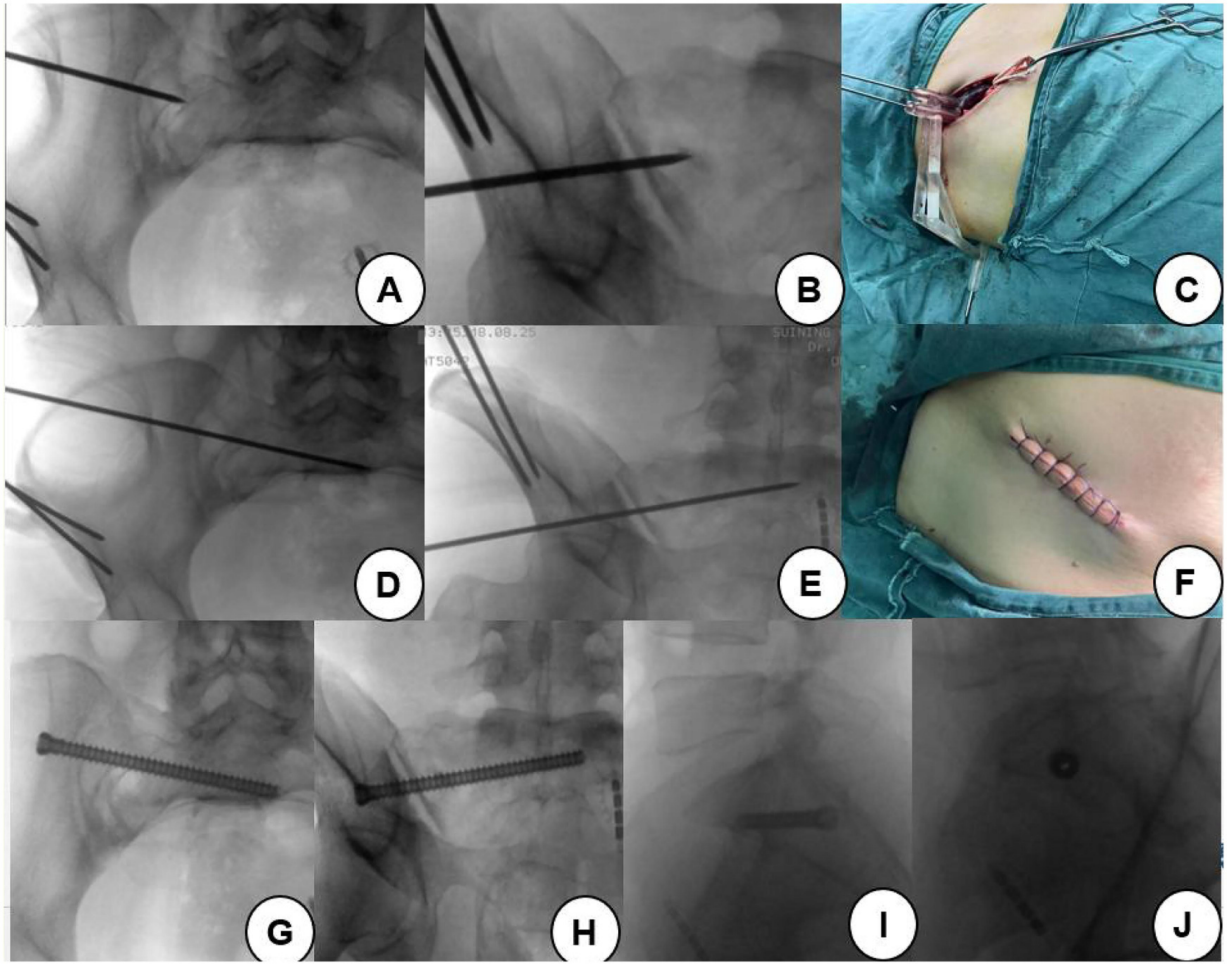
Surgical incision and intraoperative operation. **(A)** After fixing the guide plate, the position of the guide pin is determined at the entrance position. **(B)** The position of the guide pin is determined at the exit position. **(C)** Schematic diagram of the guide plate, base, and insertion of the guide needle during operation. **(D)** After being placed in a certain depth, the entrance position is irradiated to determine the position of the guide pin. **(E)** The position of the guide pin is determined by irradiation at the exit position. **(F)** The wound at the anterior superior iliac spine was sutured after the operation. **(G)** Entrance position after screw placement. **(H)** Exit position after screw placement; **(I)** Posterior radiograph of the insertion of the screw. **(J)** Screw axis bitmap after screw placement.

The surgical procedure was different in the Observation group. Traditional sacroiliac screw placement requires a prone position for insertion. Isolated posterior ring fractures are often associated with anterior ring injuries, so both the anterior and posterior rings need to be treated during surgery. For anterior ring treatment, a supine position is required; thus, intraoperatively, the patient needs to be repositioned, which increases the operation time. Using a 3D-printed guide plate for anterior ring fixation during screw placement allows for the surgery to be performed in a supine position, fixing both the anterior and posterior rings simultaneously, thereby avoiding the additional time required for patient repositioning. After admission, the preparation of the 3D-printed guide plate can immediately begin, which includes the preparation of the pelvic model, preoperative design of the guide, and fabrication and disinfection of the guide before use. The guide production takes approximately 3 h, followed by low-temperature plasma disinfection before use in the surgery. The lower abdomen and the injured hip were routinely disinfected and an incision approximately 7 cm in length in the anterior superior iliac spine of the injured side to the upper back was made. After the iliac spine was exposed, the surface of the iliac spine and the soft tissues of the iliac plate were picked up using an electric knife to fully expose the bone surface. Then, the base of the 3D-printed guide plate was attached to the iliac spine, and the iliac bone was implanted with two 2.5-mm Kirschner pins along the pre-prepared hollow channel to fully fix the iliac bone to the base. The hollow guide column at the skin surface was then used to make a 0.5 cm incision, and curved pliers were used to separate the lateral soft tissue of the sacrum along the direction of the guide column, reducing the soft tissue in the hollow guide column image. A 2.5 mm Kirschner's needle was then inserted into the guide column, 'with C-arm fluoroscopy used to verify the accuracy of the needle point and direct the Kirschner's needle to the specified position. The guide plate was then removed, followed by hollow screw reaming and placement of the 6.5-mm sacroiliac screws. The surgical incision and intraoperative operation are shown in Figure 2. INFIX was inserted anteriorly in the same manner as in the Control group, noting that the incision on the injured side could be extended slightly downward along the incision of the guide plate to expose the position of the iliac screw, and the wound was flushed and sutured.

### 2.4 Observation and efficacy evaluation indices

**Figure 3:**
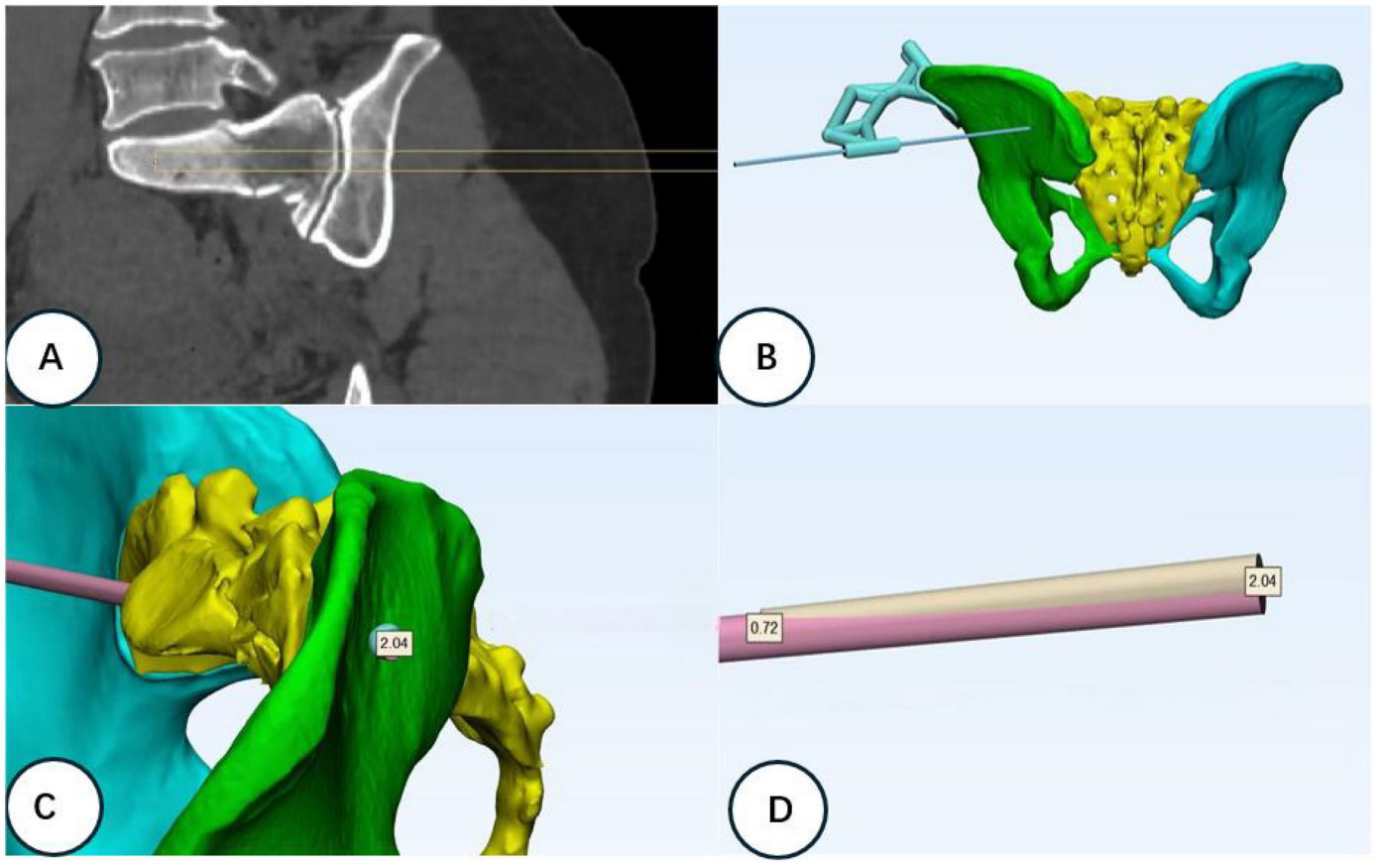
Channel design and postoperative screw position error analysis. **(A)** Schematic diagram of the ideal position of the designed channel screw according to the fracture type and pelvis model data. **(B)** Simulation of the iliac surface position after channel screw placement. **(C)** Comparison analysis of the actual channel after placement of the channel screw on the software preoperatively. The screw insertion point error is 2.04 mm. **(D)** Extraction of the designed channel and the actual channel for the error analysis of the needle exit point. The screw exit point error is 0.72 mm.

The operation time, amount of intraoperative bleeding, number of C-arm fluoroscopies, and number of guide pin adjustments during surgery for each screw implantation during surgery were recorded. The preoperative and postoperative fracture reduction was evaluated according to Matta’s criteria (15, 16), and the screw position was assessed using the Gras classification (17, 18) (see Figure 3). The patients’ postoperative functional recovery was assessed using the Majeed scoring system (19).

### 2.5 Statistical methods

Data were processed and analyzed using SPSS version 27.0. The measurement data were expressed as mean ± standard deviation ($\bar{X}\pm S$) and t-tests were used. The count data were examined using two tests. A difference was regarded as statistically significant at *P* < 0.05.

## 3 Results

Except for one patient who was lost to the study, the patients completed more than 6 months of clinical follow-up. The guide plate group was followed up for 6–12 months (8.1 ± 3.32) and the freehand group was followed up for 6–14 months (7.8 ± 4.13), and the difference between the two groups of patients was not statistically significant (*P* > 0.05). In total, 23 sacroiliac screws were inserted in the guide plate group, and 25 screws were inserted in the freehand group. The difference between the two groups was statistically significant (*P* < 0.05), with a shorter operation time, fewer intraoperative fluoroscopies, fewer adjustments of the guide pins, more intraoperative bleeding, and larger surgical incisions in the plate-guided group (Table 2).

**Table 2:** Comparison of surgical outcomes between the guide plate group and the freehand group.

Group	Time (min)	Number of adjustment screws	Intraoperative fluoroscopy time	Intraoperative bleeding volume (mL)	Incision length (cm)
Observation	82.6 ± 6.518	0.40 ± 0.681	11.65 ± 3.117	16.60 ± 5.642	8.10 ± 0.75
Control	107.25 ± 9.839	6.20 ± 3.205	48.05 ± 7.258	10.30 ± 4.354	2.78 ± 0.53
t	−4.267	−7.916	−20.608	3.953	25.920
P	<0.001	<0.001	<0.001	<0.001	<0.001

Pelvic digital radiography (DR) and CT were reviewed as soon as possible after surgery, and the offset distance between the preoperative planned screw tip position and the actual postoperative screw tip position in the two groups was denoted as the screw offset distance. The results showed that there was no statistically significant difference between the two groups in terms of their Matta’s criteria scores for fracture restoration quality and Gras classification of screw position (*P* > 0.05), and the screw offset distance in the guide plate group was smaller than that of the freehand group, with a statistically significant difference (*P* < 0.05) (Table 3).

**Table 3:** Comparison of fracture restoration quality, screw position classification, and screw offset distance between the guide plate group and the freehand group.

Group	Matta’s criteria score		Gras classification of screw position			Screw offset distance (mm)
	Excellent	Good	I	II	III	
Guide plate	17	3	16	4	0	2.73 ± 0.855
Freehand	15	5	13	5	1	3.42 ± 0.843
χ/t	0.625		1.032			−2.589
P	0.429		0.597			0.014

## 4 Discussion

Since the posterior pelvic ring accounts for 60% of pelvic stability, it is crucial for the body's weight-bearing function (20). Pelvic fractures are often caused by high-energy trauma and are frequently accompanied by posterior ring fractures. For unstable fractures, surgical intervention is typically recommended, with treatment options including anterior plate screw fixation, external brace fixation, percutaneous retrograde screw fixation, INFIX internal fixation, and posterior sacroiliac screw fixation. Traditional surgery often requires larger incisions and more soft tissue dissection, which increases intraoperative bleeding and the risk of infection and slows postoperative recovery. This has led to a growing preference for minimally invasive approaches to pelvic fracture surgery (18).

Obese patients, however, may face additional challenges. Larger incisions are needed to expose the iliac crest, which increases surgical time and the risk of wound complications. Furthermore, C-arm fluoroscopy can encounter technical limitations due to body mass, making it difficult to achieve clear and reliable imaging for screw placement. In these cases, 3D-printed guide plates offer significant advantages by reducing reliance on fluoroscopic imaging, improving screw placement accuracy, and potentially shortening surgery time. Sacroiliac screws are favored for their minimal invasiveness, reliable fixation, and comparable efficacy to traditional incisional plate screw fixation, and have become a standard approach for posterior pelvic ring fractures (21). However, the complexity of pelvic anatomy and individual anatomical variations make precise sacroiliac screw placement challenging when relying solely on repeated intraoperative C-arm fluoroscopy, increasing the risk of complications (22). As a result, there is growing interest in improving screw placement accuracy and reducing fluoroscopy use to shorten surgical time. While surgical robots and intraoperative CT offer significant benefits by improving accuracy and reducing complications, the high cost of these technologies limits their widespread adoption (23, 24). Studies have shown that 3D-printed guides can shorten surgical time, improve screw placement accuracy, and reduce the risk of vascular and nerve injury (25). In light of these challenges, 3D-printed guides hold promise for improving the surgical outcomes, particularly in patients in whom it is difficult to achieve precise screw placement. Further studies are needed to assess the effectiveness of 3D-printed guides in different patient populations, including those with obesity, to fully understand how they overcome the limitations of traditional imaging techniques.

The process of 3D printing a guide plate includes constructing a three-dimensional model of the patient's pelvis through Mimics software, simulating and designing the entry point of the sacroiliac screw, the entry angle, and screw length in the system, and using the principle of reverse thinking to make the hollow guide post, linking the device and the base of the guide plate *in vitro*. During surgery, screws can be placed directly from the hollow guide post to reduce surgical difficulty, improve guide wire placement accuracy, shorten surgical time, and reduce radiation (26). Currently, the most researched 3D-printed guides are posterior iliac wing base guides, i.e., incision of the skin and soft tissues around the posterior iliac bone in the prone position to find a specific anatomical structure site for the placement of 3D-printed guides to assist in sacroiliac screw placement close to the bone’s surface. However, a simple posterior ring fracture is rare, and the surgery often needs to fix the anterior pelvic ring, in which case the surgical position needs to be changed to the supine position to fix the anterior ring, which increases the surgical time and difficulty of the surgery. In this study, the guide plate was designed to assist the placement of sacroiliac screws in the supine position, so that both anterior and posterior pelvic ring fracture repairs could be performed in the same position, reducing the surgical time.

The results of this study demonstrate that the use of the anterior superior iliac spine 3D-printed guide plate for sacroiliac screw placement significantly shortens surgical time compared to traditional freehand placement. Previous studies have reported variable surgical times for fluoroscopy-assisted sacroiliac screw fixation. For example, a study by Zhou et al. (10) found that traditional sacroiliac screw fixation typically takes 80–100 min, while our study found an average surgical time of 60 min in the guide plate group. This reduction in surgical time highlights the advantage of 3D-printed guide plates in minimizing fluoroscopy and optimizing screw placement. It is important to note that surgical time can also be influenced by the experience and skill of the surgeon. As mentioned by You et al. (11), experienced surgeons may complete freehand placement faster due to familiarity with pelvic anatomy. However, 3D-printed guides are particularly beneficial for less experienced surgeons, as they reduce reliance on fluoroscopy and help improve precision. Moreover, patient factors, such as body mass index and fracture complexity, can also impact surgical time. Zhou et al. showed that in obese patients, larger incisions and additional fluoroscopy increased surgical time. Future studies should further explore the influence of these factors on surgical time when using 3D-printed guides. Although the surgical incision length and amount of intraoperative bleeding in our study were less favorable than in freehand placement, the overall reduction in surgical time, the number of radiographs, and improved precision make the 3D-printed guide approach a more beneficial option for patients.

Most sacral fractures are fixed with sacroiliac screws, with the bone surface entry point located in the iliac wing area, where there are important structures such as the superior gluteal artery, nerves, and gluteal muscles. The traditional posterior application of bone guides requires stripping the soft tissues in this area and near the posterior superior iliac spine, which involves risks such as damage to important structures (11). In this study, we chose a specific anatomical location near the anterior superior iliac spine, which is clearly positioned on the body surface and has few soft tissue structures, as the 3D printing guide plate base attachment site. This avoids extensive stripping of the buttocks and theoretically reduces the risk of injury to important structures in the posterior buttocks. The design of the guide plate base makes full use of the three-dimensional structure of the anterior superior iliac spine, iliac rim, and external iliac wing. By making an incision of comparable size to the guide plate in the direction of the iliac spine at the anterior superior iliac spine site, the tissues are separated to expose the anterior superior iliac spine, part of the iliac rim, and the external iliac plate. The base of the guide plate fits with the anterior superior iliac spine anteriorly, the iliac rim superiorly, and the external surface with the external plate of the ilium, and the three planes together make the guide plate different from the single-bone-surface-fit bone guide plate base in previous studies, with a higher bone-surface fit. The connecting device for the 3D-printed guide plate has a large span and a certain amount of deformation of the material due to the curved arc from the anterior superior iliac spine to the lateral side of the buttock. The connection device designed in this study abandons the simple structure of the single-rod arc connection and adopts the double-rod connection. Utilizing the principle that triangles have stability, multiple triangles were added between the double-rod structures to increase the stability of the connection device.

The ability of the 3D-printed guide plate to be accurately anchored in the preoperative design position and to maintain stability in the direction of needle advancement during assisted screw placement is critical to ensure accurate screw placement (29). Guide wire placement accuracy can be improved during the design and intraoperative manipulation of the guide plate in the following ways. (1) Because the sacroiliac screw placement point for pelvic fractures is located near the iliac wing, which is relatively flat, the tip of the guide pin slips on the bone surface during the placement process, and there is a point of entry error, which may lead to the failure of the screw placement; therefore, when designing the guide plate’s hollow guide post, the hollow guide post should be perpendicular to the bone surface as much as possible to minimize slippage of the point of entry. (2) Thus, a sharp Kirschner needle is recommended to reduce the occurrence of the bone surface slippage phenomenon. (3) When designing the hollow guide posts of the guide plate, the system should ensure that the cannulated screws are located in the bone in the coronal, sagittal, and transverse planes in the three-dimensional software, so as to reduce the difficulty of intraoperative C-arm fluoroscopic assessment. (4) Before placing the guide plate, the muscle tissue of the anterior superior iliac spine, iliac rim, and external plate of the ilium should be removed as much as possible, so that the base of the guide plate can completely adhere to the bone surface; furthermore, the base of the guide plate should be completely flush with the case of the electric drill when drilling into the base of the two crossed Kirschner's pins to avoid the slight displacement of the base of the guide plate, which could result in a large deviation of the point of entry of the hollow screws. (5) To prevent the guide needle from deviating due to interference from abundant soft tissue, a hollow guide post can be used to create a subcutaneous tunnel. This tunnel guides the needle directly to the bone surface, minimizing placement error.

With the increasing awareness of surgical scars among patients, especially those located on the iliac crest, which may affect daily activities such as wearing trousers and belts, the length of the surgical incision and its esthetic outcome have become particularly important. The use of 3D-printed guide plate technology not only improves surgical precision, reduces operative time, and minimizes the number of fluoroscopic exposures, but also effectively reduces the incision length, avoiding excessive soft tissue dissection. By precisely designing the screw insertion path, the incision size is minimized, thereby reducing postoperative discomfort, enhancing esthetic outcomes, and meeting patients’’ expectations regarding the appearance of scars, ultimately improving postoperative quality of life.

It is crucial to recognize the limitations of this design study. (1) The additional cost of preoperative design and pelvic modeling for the patient, the production of the guide plate, and the intraoperative surgical incision at the anterior superior iliac spine site (which brings about problems such as increased bleeding and increased chances of wound infections) increase the trauma and financial burden for the patient. However, the accurate use of the guide plate saves surgical time for both the patient and the doctor, reduces the number of intraoperative fluoroscopies, and decreases the risk of the surgery, which is beneficial to the patient as a whole. Thus, the technique is favorable to the patient overall. (2) In previous studies, the posterior superior iliac spine and the iliac wing were used as bone guides, with the possibility of intraoperative damage to important structures; in this study, we used the anterior superior iliac spine bone guide, with the possibility of damage to the lateral femoral cutaneous nerve when exposing the anterior superior iliac spine during the operation. Intraoperative incisions were made to free the lateral femoral cutaneous nerve when necessary. Since all the patients in this study underwent INFIX internal fixation of the anterior ring while fixing the posterior ring, and the INFIX entry point is located below the anterior superior iliac spine, the incision on the affected side can be extended downward to expose the INFIX entry point during the intraoperative operation, which provides the possibility of anatomical freeing of the lateral femoral cutaneous nerve and reduces the possibility of injury to the lateral femoral cutaneous nerve. (3) This study included cases with sacral fractures and sacroiliac joint dislocations that were not severe. For patients with severe displacement, whether to adopt the preoperative simulation of reset method, using the principle of pelvic mirroring and applying the data from the healthy side to design the sacroiliac screw channel, is the focus of our next study. (4) The number of cases in this study was small, and included patients with non-severe fracture displacement, so there was selection bias to a certain extent, and the sample size needs to be increased in subsequent studies. (5) This study did not include a sufficient number of obese patients, who may face unique challenges during surgery. Obese individuals typically require larger incisions to achieve adequate exposure of the iliac crest, which could increase surgical time, intraoperative bleeding, and the risk of infection. Moreover, C-arm fluoroscopy may be less effective in obese patients due to body mass, leading to difficulties in achieving optimal screw placement. The use of 3D-printed guide plates may prove especially beneficial in these cases, as they can reduce reliance on fluoroscopic imaging, improve screw placement accuracy, and shorten the surgical time. However, this study', which focused on patients with normal or slim body types, did not allow for a sufficient exploration of these issues. Future studies should include a larger sample of obese patients to assess the effectiveness and potential advantages of 3D-printed guide plates in this population, particularly in overcoming the limitations of traditional imaging techniques and improving surgical outcomes.

In summary, the 3D-printed guide plate for placement of sacroiliac screws in the anterior superior iliac spine designed in this study had comparable clinical efficacy with the traditional freehand technique, and had the advantages of shorter surgical time, fewer intraoperative fluoroscopies, and higher accuracy. The 3D-printed guide plate designed in this study allows the surgeon to complete anterior and posterior ring fixation at the same time in the supine position, which removes the requirement for an intraoperative position change, simplifies the surgical process, and shortens the surgical time compared with the posterior iliac bone guide plate. This study had a small sample size and certain limitations, and future studies should be optimized and have a larger sample size and long-term follow-up to further verify this 3D-printed guide plate’s advantages.

## Data Availability

The raw data supporting the conclusions of this article will be made available by the authors, without undue reservation.

## References

[1] Wu K Bai X Li S. A new individually designed 3D printing guide plate-assisted internal fixator (INFIX) surgery for the treatment of pelvic fractures. J Orthop Surg Res. (2025) 20(1):770. 10.1186/s13018-025-06125-z 40830964 PMC12366166

[2] Al-Naseem A Sallam A Gonnah A Masoud O Abd-El-Barr MM Aleem IS. Robot-assisted versus conventional percutaneous sacroiliac screw fixation for posterior pelvic ring injuries: a systematic review and meta-analysis. Eur J Orthop Surg Traumatol. (2023) 33(1):9–20. 10.1007/s00590-021-03167-x 34842991

[3] Huang S He S Kenmegne GR Yin Y Yu Y Fang Y. Anterior subcutaneous internal fixator (INFIX) versus plate fixation for anterior ring injury in tile C pelvic fractures: a retrospective study. BMC Surg. (2025) 25(1):110. 10.1186/s12893-025-02844-6 40119294 PMC11927202

[4] Abdelfattah A Moed BR. Ligamentous contributions to pelvic stability in a rotationally unstable open-book injury: a cadaver study. Injury. (2014) 45(10):1599–603. 10.1016/j.injury.2014.05.026 24938676

[5] Sichting F Rossol J Soisson O Klima S Milani T Hammer N. Pelvic belt effects on sacroiliac joint ligaments: a computational approach to understanding the therapeutic effects of pelvic belts. Pain Physician. (2014) 17(1):43–51. 10.36076/ppj.2014/17/43 24452644

[6] Liu F Lei Q Cai L Jiang M Yang H Wang K Efficacy comparison between iliosacral screw fixation of the posterior pelvic ring fracture with the assistance of modified percutaneous three-dimensional printing guide template and conventional fluoroscopy. Zhong Nan Da Xue Xue Bao Yi Xue Ban. (2023) 48(11):1703–10. 10.11817/j.issn.1672-7347.2023.230122 38432861 PMC10929951

[7] Santoro G Braidotti P Gregori F Santoro A Domenicucci M. Traumatic sacral fractures: navigation technique in instrumented stabilization. World Neurosurg. (2019) 131:399–407. 10.1016/j.wneu.2019.07.050 31658582

[8] Shetty AP Renjith KR Perumal R Anand SV Kanna RM Rajasekaran S. Posterior stabilization of unstable sacral fractures: a single-center experience of percutaneous sacroiliac screw and lumbopelvic fixation in 67 cases. Asian Spine J. (2021) 15(5):575–83. 10.31616/asj.2020.0337 33355847 PMC8561155

[9] Suero EM Greiner A Becker CA Cavalcanti Kußmaul A Weidert S Pfeufer D Biomechanical stability of sacroiliac screw osteosynthesis with and without cement augmentation. Injury. (2021) 52(10):2707–11. 10.1016/j.injury.2020.01.043 32033807

[10] Zhou W Xia T Liu Y Cao F Liu M Liu J Comparative study of sacroiliac screw placement guided by 3D-printed template technology and x-ray fluoroscopy. Arch Orthop Trauma Surg. (2020) 140(1):11–7. 10.1007/s00402-019-03207-6 31127408 PMC6942002

[11] You MR Fan ZQ Ye HM Wang Z Zou CH Dong XP. The design and application of individualized 3D printing-assisted guide plates in assisting sacroiliac screw insertion. Comput Assist Surg (Abingdon). (2022) 27(1):113–9. 10.1080/24699322.2022.2102542 35867539

[12] Tao X Lyu F Sugand K Zhou K Wang H. Does a novel 3D printed individualized guiding template based on cutaneous fiducial markers contribute to accurate percutaneous insertion of pelvic screws? A preliminary phantom and cadaver study. BMC Surg. (2024) 24(1):105. 10.1186/s12893-024-02402-6 38614998 PMC11015658

[13] Mitsuizawa S Kusakabe K Matsuda S. Minimally invasive transiliac anatomical locking plate for posterior pelvic ring injury: a technical trick of the gull wing plate. J Clin Orthopaedic Trauma. (2022) 33:101991. 10.1016/j.jcot.2022.101991 PMC9436802 36061970

[14] Chen K Yao S Yang F Drepaul D Telemacque D Zhu F Minimally invasive screw fixation of unstable pelvic fractures using the “blunt end” Kirschner wire technique assisted by 3D printed external template. Biomed Res Int. (2019) 2019:1524908. 10.1155/2019/1524908 31772932 PMC6854157

[15] Liang B Chen Q Liu S Chen S Yao Q Wei B A feasibility study of individual 3D-printed navigation template for the deep external fixator pin position on the iliac crest. BMC Musculoskelet Disord. (2020) 21(1):478. 10.1186/s12891-020-03509-6 32693799 PMC7372844

[16] Matta JM. Operative treatment of acetabular fractures through the ilioinguinal approach: a 10-year perspective. J Orthop Trauma. (2006) 20(1 Suppl):S20–9. 10.1097/01.bot.0000202389.40246.c0 16385203

[17] Gras F Marintschev I Wilharm A Klos K Mückley T Hofmann GO. 2D-fluoroscopic navigated percutaneous screw fixation of pelvic ring injuries—a case series. BMC Musculoskelet Disord. (2010) 11:153. 10.1186/1471-2474-11-153 20609243 PMC2916892

[18] Yu T Cheng XL Qu Y Dong RP Kang MY Zhao JW. Computer navigation-assisted minimally invasive percutaneous screw placement for pelvic fractures. World J Clin Cases. (2020) 8(12):2464–72. 10.12998/wjcc.v8.i12.2464 32607323 PMC7322419

[19] Majeed SA. Grading the outcome of pelvic fractures. J Bone Joint Surg Br Vol. (1989) 71(2):304–6. 10.1302/0301-620X.71B2.2925751 2925751

[20] Jing Y Chang L Cong B Wang J Chen M Tang Z Preoperative 3D printing planning technology combined with orthopedic surgical robot-assisted minimally invasive screw fixation for the treatment of pelvic fractures: a retrospective study. Peer J. (2024) 12:e18632. 10.7717/peerj.18632 39677955 PMC11646416

[21] Timmer RA Verhage SM Krijnen P Meylaerts SAG Schipper IB. Indications for surgical fixation of low-energy pelvic ring fractures in elderly: a systematic review. Arch Orthop Trauma Surg. (2023) 143(5):2417–28. 10.1007/s00402-022-04438-w 35462589 PMC10110636

[22] Chon CS Jeong JH Kang B Kim HS Jung GH. Computational simulation study on ilio-sacral screw fixations for pelvic ring injuries and implications in Asian sacrum. Eur J Orthop Surg Traumatol. (2018) 28(3):439–44. 10.1007/s00590-017-2061-2 29027586

[23] Yang Z Sheng B Liu D Chen X Guan R Wang Y Intraoperative CT-assisted sacroiliac screw fixation for the treatment of posterior pelvic ring injury: a comparative study with conventional intraoperative imaging. Sci Rep. (2022) 12(1):17767. 10.1038/s41598-022-22706-y 36273094 PMC9588013

[24] Wang J Zhang T Han W Hua K Wu X. Robot-assisted S2 screw fixation for posterior pelvic ring injury. Injury. (2023) 54(Suppl 2):S3–7. 10.1016/j.injury.2020.11.044 33317816

[25] Yang Z Sheng B Liu D Wang Y Liu C Xiao R. Sacroiliac screw fixation navigated with three-dimensional printing personalized guide template for the treatment of posterior pelvic ring injury: a case report. Front Surg. (2022) 9:1025650. 10.3389/fsurg.2022.1025650 36684191 PMC9852618

[26] Zhang YW Xiao X Xiao Y Chen X Zhang SL Deng L. Efficacy and prognosis of 3D printing technology in treatment of high-energy trans-syndesmotic ankle fracture dislocation—“log-splitter” injury. Med Sci Monit. (2019) 25:4233–43. 10.12659/MSM.916884 31172985 PMC6572869

[27] Ivanov S Valchanov P Hristov S Veselinov D Gueorguiev B. Management of complex acetabular fractures by using 3D printed models. Medicina (Kaunas). (2022) 58(12):1854. 10.3390/medicina58121854 36557056 PMC9785751

